# Pepsin

**Published:** 1874-05

**Authors:** R. T. Edes


*BOSTON MEDICAL AND SURGICAL JOURNAL—February*, 1874.

Pepsin.—*By R. T. Edes, M.D.*—Much of the dissatisfaction with pepsin expressed by physicians is due to the use of preparations which contain little or none of it.

The pepsin made by Schæffer's process is by far superior to any other in ordinary use.

The wine is feeble, but not necessarily inert.

Elixirs of pepsine and bismuth are humbugs.

Pepsin should be administered with an acid, and with as few drugs as possible. A small amount of alcohol is not inadmissible, but a large amount retards digestion.

Its beneficial action is not limited by the amount of albumen which it dissolves in a test tube without change or renewal of any of the contents.

